# Draft Genome Sequences of Six *Bacillus* Strains and One *Brevibacillus* Strain Isolated from the Rhizosphere of Perennial Ryegrass (Lolium perenne)

**DOI:** 10.1128/MRA.01586-18

**Published:** 2019-01-24

**Authors:** Zhibo Li, Chunxu Song, Anne de Jong, Oscar P. Kuipers

**Affiliations:** a Department of Molecular Genetics, University of Groningen, Groningen, the Netherlands; Georgia Institute of Technology

## Abstract

Seven strains of endospore-forming bacteria with antagonistic activity against a series of plant pathogens were sequenced in order to investigate their antimicrobial gene clusters and antimicrobial modes of action. The selected strains include six *Bacillus* strains and one *Brevibacillus* strain.

## ANNOUNCEMENT

Plant diseases are major yield-reducing and quality-limiting factors in the production of food. More than 50,000 pathogenic species of microorganisms are invasive to crops and cause a yield reduction of about 7.5% (1). To control crop pathogens, many chemical pesticides have been developed and applied, resulting in serious environmental problems. Hence, there is an urgent need to develop sustainable approaches to control crop pathogens. The application of beneficial microbes has been reported to be effective in pathogen control and is environmentally friendly (2, 3). A well-known beneficial genus of bacteria is *Bacillus*, the species of which are Gram positive, rod shaped, spore forming, and widely distributed in the environment. *Bacillus* is able to produce an array of antimicrobial compounds, including surfactins, fengycins, and iturins, which have strong inhibitory activity on different pathogens (4). The ability to form endospores under biotic and abiotic conditions makes various species of *Bacillus* promising commercial biocontrol agents.

Seven *Bacillus*-like biocontrol strains were isolated from the rhizosphere of healthy perennial ryegrass in Groningen, the Netherlands. Briefly, 1 g of rhizosphere soil sample was suspended in 9 ml of 10 mM MgSO_4_ solution. After heating at 80°C for 10 min, the soil solution was diluted 1,000 times with 10 mM sterilized MgSO_4_ solution and spread onto Lennox broth agar (Formedium) plates. The plates were incubated at 28°C for 24 h to obtain single colonies.

For genome sequencing, a single colony of each strain was grown in 3 ml Lennox broth (Formedium) at 28°C. Overnight cultures were diluted 50-fold in fresh LB broth and grew until the late exponential growth phase. Cells were collected by centrifugation at 10,000 rpm for 2 min, and total DNA was isolated with a GenElute bacterial genomic DNA kit (Sigma-Aldrich) according to the manufacturer’s protocol. The draft genomes were determined at GATC Biotech (Germany) with an Illumina HiSeq sequencing system. A total of 5 million paired reads (150 bp) were generated. FastQC version 0.11.5 (https://www.bioinformatics.babraham.ac.uk/projects/fastqc/) was used to examine the quality of the reads, and low-quality reads were removed with Trimmomatic version 0.38 (5). The reads were assembled *de novo* using SPAdes version 3.11.1 with default parameters (6). All of the genomes sequenced exceeded 150× coverage, and the characteristics of the obtained assemblies and genome features are described in Table 1. The genomes were annotated with the Rapid Annotations using Subsystems Technology (RAST) server (7). Strains were identified to be *Bacillus* and *Brevibacillus* species (Table 1) by phylogenetic analysis with available whole-genome sequences (our unpublished data). Genome mining was conducted with BAGEL4 (8) and antiSMASH (9), showing various potential novel bacteriocins, nonribosomal peptides (NRPs), and polyketides (PKs) in all seven strains. The exact characteristics and functions of these antimicrobial compounds are under investigation.

**TABLE 1:** Genome features and GenBank accession numbers of the strains

Strain	Genome size (kb)	G+C content (%)	*N*_50_ (kb)	No. of contigs	GenBank accession no.
Bacillus subtilis MG27	4,188	43.4	1,048	13	QJJA00000000
Bacillus velezensis MG33	4,213	45.7	342	31	QJJB00000000
Bacillus velezensis MG43	3,923	46.5	882	23	QJJC00000000
Bacillus pumilus MG52	3,804	41.6	212	39	QJIZ00000000
Brevibacillus laterosporus MG64	5,153	40.7	130	103	QJJD00000000
Bacillus altitudinis MG75	3,865	41.0	248	42	QIMF00000000
Bacillus pumilus MG84	3,657	41.5	167	33	QJJE00000000

### Data availability.

The genome sequences of the seven potential biocontrol strains have been deposited in DDBJ/EMBL/GenBank under the accession numbers QJJA00000000, QJJB00000000, QJJC00000000, QJIZ00000000, QJJD00000000, QIMF00000000, and QJJE00000000. The versions described in this paper are the first versions, QJJA01000000, QJJB01000000, QJJC01000000, QJIZ01000000, QJJD01000000, QIMF01000000, and QJJE01000000. The raw reads were submitted to the Sequence Read Archive under the accession numbers SRR8305992, SRR8316559, SRR8316560, SRR8316751, SRR8316749, SRR8316750, and SRR8316752.
